# Supplementary material for: HCV universal EHR prompt successfully increases screening, highlights potential disparities
Source: PLoS One. 2023 Mar 2;18(3):e0279972. doi: 10.1371/journal.pone.0279972 (PMC9980726; doi:10.1371/journal.pone.0279972)
Supplement: Attachment — Submitted filename: Response to Reviewers.docx [file pone.0279972.s003.docx]

**Comment:** Please include a reference for micro-elimination (Lines 75-76).

**Response:** An appropriate reference was added for this statement.

**Comment:** Lines 136-137:  I realize authors may be rounding, however, I think the text and table should be the same- is the percent 79.8% or 80%?

**Response:** The percent was changed to 79.8.

**Comment:** Please ensure that your manuscript meets PLOS ONE's style requirements, including those for file naming. The PLOS ONE style templates can be found at 
<https://journals.plos.org/plosone/s/file?id=wjVg/PLOSOne_formatting_sample_main_body.pdf> and 
<https://journals.plos.org/plosone/s/file?id=ba62/PLOSOne_formatting_sample_title_authors_affiliations.pdf>

**Response:** The manuscript has been updated to reflect all style requirements.

**Comment:** Please include your ethical information in the Methods section of your manuscript

**Comment:** Please amend your current ethics statement to address the following concerns:
a) Did participants provide their written or verbal informed consent to participate in this study?
b) If consent was verbal, please explain i) why written consent was not obtained, ii) how you documented participant consent, and iii) whether the ethics committees/IRB approved this consent procedure.

**Response:** The ethical information statement was moved from the statistical analysis subsection of the methods to the extraction and variables subsection. The statement was amended to include information regarding consent.

**Comment:** Thank you for stating the following in the Competing Interests section:

I have read the journal's policy and the authors of this manuscript have the following competing interests: Dawn Fishbein, MD receives grant funding for this research from Gilead Sciences, Inc through her employer. She has stock ownership in Abbvie and Merck is a discretionary portfolio with a value less than $10,000.

Please confirm that this does not alter your adherence to all PLOS ONE policies on sharing data and materials, by including the following statement: "This does not alter our adherence to  PLOS ONE policies on sharing data and materials.” (as detailed online in our guide for authors <http://journals.plos.org/plosone/s/competing-interests>).  If there are restrictions on sharing of data and/or materials, please state these. Please note that we cannot proceed with consideration of your article until this information has been declared.

Please include your updated Competing Interests statement in your cover letter; we will change the online submission form on your behalf.

**Response:** The cover letter has been updated to reflect this requirement.

**Comment:** We note that you have stated that you will provide repository information for your data at acceptance. Should your manuscript be accepted for publication, we will hold it until you provide the relevant accession numbers or DOIs necessary to access your data. If you wish to make changes to your Data Availability statement, please describe these changes in your cover letter and we will update your Data Availability statement to reflect the information you provide.

**Response:** We are no longer able to provide a repository of our data. We can provide the PLOS ONE statisticians with a de-identified data set if requested and as long as it is confidential.

**Comment:** Please include your full ethics statement in the ‘Methods’ section of your manuscript file. In your statement, please include the full name of the IRB or ethics committee who approved or waived your study, as well as whether or not you obtained informed written or verbal consent. If consent was waived for your study, please include this information in your statement as well.

**Response:** See above response regarding ethics statement.

**Comment:** Please review your reference list to ensure that it is complete and correct. If you have cited papers that have been retracted, please include the rationale for doing so in the manuscript text, or remove these references and replace them with relevant current references. Any changes to the reference list should be mentioned in the rebuttal letter that accompanies your revised manuscript. If you need to cite a retracted article, indicate the article’s retracted status in the References list and also include a citation and full reference for the retraction notice.

**Response:** The reference list has been updated to reflect the correct articles which were cited. No retracted papers were cited, however several references from an earlier version of the paper were included and have now been removed.

**Comment:** First, the readers need more information about the health system mentioned. All outpatient visits are not the same, so some broad breakdown of visits for primary care, specialty care or ED would be helpful. This is also germane to the subgroups the authors mention with disparities, as some specialty physicians may not take ownership of screening when they see this as the purview of the primary care physicians. Also, a general breakdown of patients seen within the system (race, ethnicity, insurance breakdown) would be helpful to better understand how the screened population differs from all patients seen.

**Response:** Unfortunately, the way that our health system collects data only allows us to differentiate emergency visits from other type of outpatient visits; although we did consider filtering out specialty from primary care, this was not possible. Given the importance of this differentiation, we amended the limitations to recognize the absence of this data.

We did categorize the population by demographics comparing screened and un-screened in the shorter period surrounding the EHR alert, see Table 2. It was not feasible to break down screened vs. unscreened demographics for the entire 48-month period, as was discussed in the limitations.

**Comment:** Second, there is no discussion of what education and/or preparation was provided prior to initiation. Were providers given a primer as to the policy changes that led to the BPA? Some physicians are very aware while others may be oblivious. We need more information to help understand the impact of these changes.

**Response:** No education or preparation was provided specifically for this new EHR alert. There was an announcement within an email of clinical updates that was sent out to the entire physician/APP group. The EHR alert itself was the principal method of disseminating this new guideline; otherwise, it was the responsibility of providers/practices to remain updated on the recommendations.

**Comment:** The authors state that more women were screened. Given that universal screening in pregnancy was a key inclusion in both public health recommendations, with the CDC recommending screening with every pregnancy, this is expected. The authors neglect to mention this subgroup in their results and it is a significant omission. Some additional discussion of pregnancy screening needs to be included.

**Response:** Another branch of our research division is actively working on projects looking at Hep C in pregnancy. Unfortunately, we did not extract this data when we began our analysis and therefore do not have It available for further analysis of this cohort in the context of this study. Given the importance of this population, we amended the limitations to recognize the absence of this data.

**Comment:** The paper would benefit from some discussion about how the authors are planning to improve the use and function of the BPA. Aside from drawing attention to the disparate practices in screening different groups, how do we get to 100%?

**Response:** As you mentioned, the main plan we propose for improving screening is focusing on who we’re missing, namely socioeconomically disadvantaged patients. In regard to specific improvements, this is more in the realm of physician bias training and education which is beyond the scope of our paper.
